# Plate fixation versus intramedullary nail or Knowles pin fixation for displaced midshaft clavicle fractures: A meta-analysis of randomized controlled trials

**DOI:** 10.1097/MD.0000000000022284

**Published:** 2020-09-25

**Authors:** Lang Li, Xiaodong Yang, Fei Xing, Jun Jiang, Xueyang Tang

**Affiliations:** a Department of Pediatric Surgery; b Department of Orthopedics, West China Hospital, Sichuan University, Chengdu, Sichuan, China.

**Keywords:** clavicle, intramedullary, meta-analysis, plate

## Abstract

**Background:**

Plate fixation and intramedullary nail/Knowles pin fixation methods are commonly used to treat displaced midshaft clavicle fractures. However, the differences between these 2 methods are unclear.

**Objective:**

This meta-analysis aimed to compare plate fixation and intramedullary nail/Knowles pin fixation for displaced midshaft clavicle fractures.

**Methods:**

We searched PubMed, EBM reviews, and Ovid Medline online for studies related to comparison of plate fixation versus intramedullary nail/Knowles pin fixation for displaced midshaft clavicle fracture from inception to June 30, 2019. Relevant literature search, data extraction, and quality assessment will be performed by 2 researchers independently. The methodological quality of all included studies was appraised using the Cochrane system for randomized trials. The RevMan 5.2 software was used for heterogeneity assessment, generating funnel-plots, data synthesis, sensitivity analysis, and determining publication bias. The fixed-effects or random-effects model was used to calculate mean difference (MD)/relative risks (RRs) and 95% confidence intervals (CIs).

**Results:**

This meta-analysis included 839 patients from 12 randomized controlled trials. We found that compared to plate fixation, intramedullary nail/Knowles pin fixation yielded a higher shoulder constant score [MD = −2.43, 95% CI (−3.46 to −1.41), *P* < .00001] and lower disabilities of the arm, shoulder and hand (DASH) score [MD = 2.98, 95% CI (0.16–5.81), *P* = .04], and lower infection rates [RR = 2.05, 95% CI (1.36–3.09), *P* = .003], operation time [MD = 20.20, 95% CI (10.80–29.60), *P* < .0001], incision size [MD = 6.09, 95% CI (4.54–7.65), *P* < .00001], and hospital stay [MD = 1.10, 95% CI (0.56–1.64), *P* < .00001] but with a higher removal rate [RR = 0.52, 95% CI (0.41–0.65), *P* < .00001] compared to plate fixation. There were no significant differences in nonunion, reintervention, or revision and refracture between these two methods. The limitation is that many studies did not demonstrate the random generated details, and only English articles were enrolled in this meta-analysis.

**Conclusions:**

Intramedullary nail/Knowles pin fixation might be an optimum choice for treating displaced midshaft clavicle fractures, with similar performance in terms of the nonunion, reintervention, or revision and refracture, and better shoulder constant and DASH scores, infection rates, and operative parameters.

## 1 Introduction

Approximately 80% of all clavicle fractures commonly reported in adults are concentrated in the middle (midshaft) of the clavicle.^[1]^ Displaced midshaft clavicle fractures are managed using conventional, nonsurgical treatments in the past.^[2,3]^ Recently, there has been a shift toward surgical treatments, enabling a reduction in nonunion and malunion, with improved shoulder function.^[4–6]^ Thus, surgical treatment has become a popular option for displaced midshaft clavicle fractures.

Both plate and intramedullary nail/Knowles pin fixation have been commonly used as surgical treatments.^[7,8]^ However, plate and intramedullary nail fixation have different characteristics.^[9]^ Although several retrospective and randomized controlled trial (RCTs) studies have compared plate and intramedullary nail fixation, the optimal treatment method remains controversial.^[10–14]^ In addition, some systematic reviews have reported the safety and effectiveness of plate and intramedullary nail/Knowles pin.^[15–18]^ However, Zhang et al^[15]^ reviewed only 4 RCTs and Hussain et al^[19]^ compared 7 RCTs and 3 quasirandomized trials, but we found that the complications were divided into 2 major categories, those requiring or not requiring surgery. Therefore, it was impossible to conclude the differences in complications between the 2 treatment methods. In addition, the removal rate has not been reported in previous systematic reviews or meta-analysis.^[19,20]^

In order to know more about 2 methods, the objective of this meta-analysis was to compare the shoulder constant score; disabilities of the arm, shoulder, and hand (DASH) score; complications; operation time; incision size; hospital stay; and removal rate of plate and intramedullary/Knowles pin fixation for the RCTs of displaced midshaft clavicle fractures in patients.

## 2 Methods

### 2.1 Eligibility criteria and literature search

**Figure 1:**
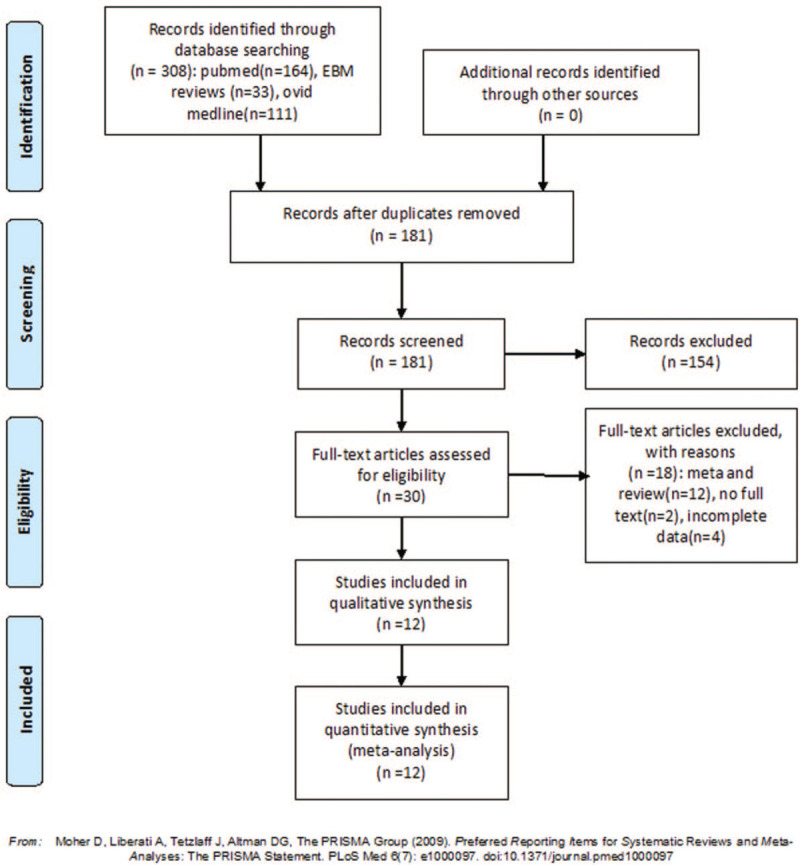
Flow chart of the literature.

This study was performed in accordance with the Preferred Reporting Items for Systematic Reviews and Meta-Analyses (PRISMA) statement.^[21]^ The process of article selection is shown in Figure 1. We searched PubMed, EBM, and Ovid Medline online from inception to June 30, 2019, using the medical subject heading (MESH): clavicle, clavicular, plate, plating, pin, intramedullary, and Knowles pin. All studies related to comparison of plate and intramedullary nail/Knowles pin fixation for displaced midshaft clavicle fractures were screened. The bibliographies and citations of each relevant article were reviewed to ensure that no article was missed. As a secondary analysis of the original research, the ethical approval was not necessary for this study.

**I**nclusion criteria for this study were patients (age >16 years) diagnosed with displaced midshaft clavicle fracture; intervention: patients treated with plate fixation; comparison treatment: patients treated with intramedullary nail/Knowles pin; outcome: related studies reported operation time, hospital time, shoulder constant score, DASH score, removal rate, and complications; study design: only RCTs were included; and language limited to English. Exclusion criteria were systematic review, case report, repeated published study, and retrospective and prospective cohort studies; studies without full-text available; and presence of pathological fractures.

### 2.2 Outcome of interest

The primary outcomes of this meta-analysis were shoulder constant score, DASH score, and complications. We divided complications into 4 categories: nonunion, reintervention or revision, refracture, and infection. The secondary outcomes were operation time, incision size, hospital stay, and removal rate.

### 2.3 Data extraction and quality assessment

All data were extracted independently by 2 reviewers according to the selection criteria (LL and FX), any disagreements were discussed and documented. Data on design type, age, sex sample size, length of follow-up, interventions, and outcomes of interest were independently extracted by 2 researchers. For quality assessment of included studies, the Cochrane system^[22]^ was used. Selection bias (random sequence generation and allocation concealment), performance bias (blinding of participants and personnel), detection bias (blinding of outcome assessment), attrition bias (incomplete outcome data), and reporting bias (selective reporting) and other sources of bias were used to evaluate the quality of included studies.

### 2.4 Statistical analysis

The RevMan software (Version 5.2, The Nordic Cochrane Centre, The Cochrane Collaboration, 2013) was used for meta-analysis and determining publication bias. For continuous variables, the MD and 95% confidence intervals (CIs) were reported. For dichotomous variables, the relative risk (RR) and 95% CI were reported. If the heterogeneity of meta-analysis results was small (*I*^2^ < 50%), the fixed effect model was used. If *I*^2^ > 50%, the random effect model was used. Sensitivity analysis was also conducted when *I*^2^ > 50%. Publication bias was also evaluated using the RevMan software. *P* values of <.05 were considered statistically significant.

## 3 Results

### 3.1 Characteristics and methodological quality of included studies

**Figure 2:**
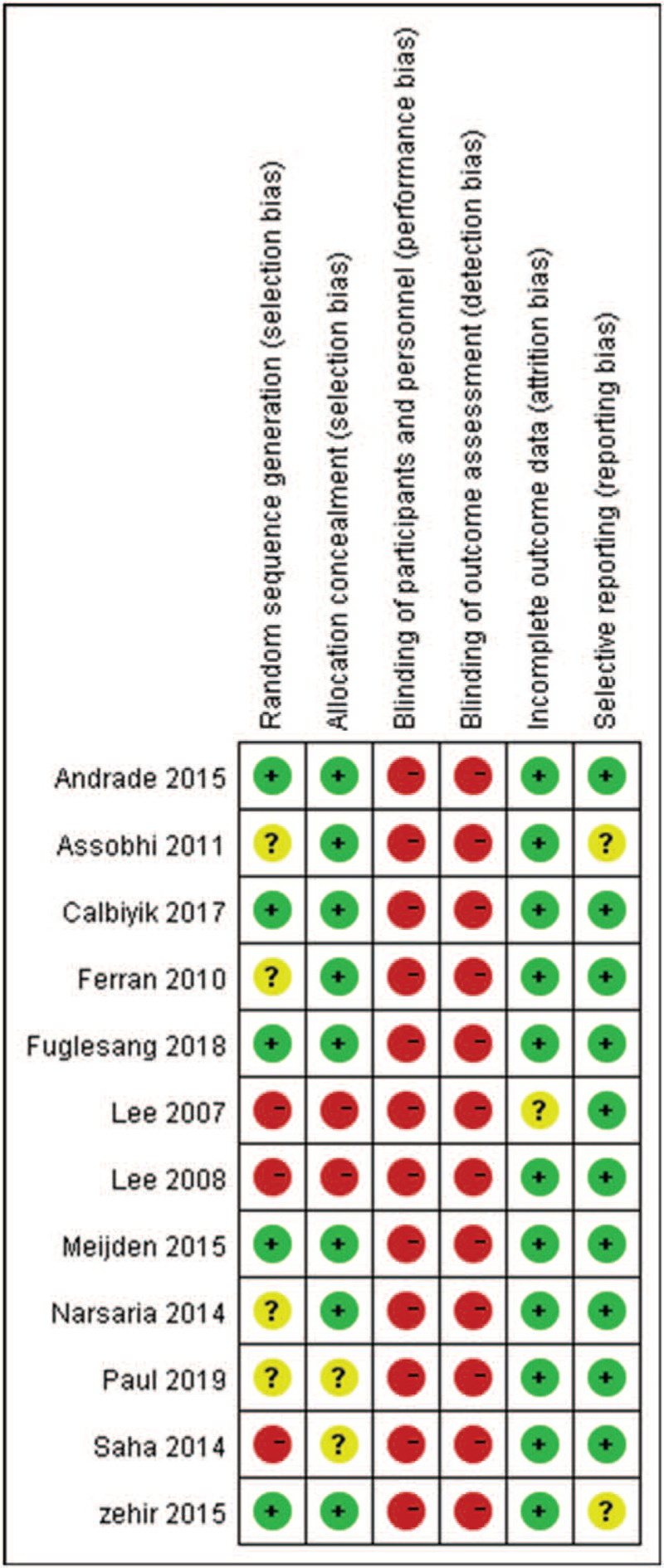
Summary of the bias risk of included studies.

The search strategy, according to PRISMA, is shown in Figure 1. After screening 308 studies, 12 RCTs^[23–34]^ that enrolled 839 patients with displaced midshaft clavicle fractures were included, the characteristics of included studies are shown in Table 1. Five studies^[23,24,30,32,34]^ reported the random sequence generation, 8 studies^[23,24,26,30–34]^ reported allocation concealment, no studies reported blinding of participants and personnel and outcome assessment, 11 studies^[23–26,28–34]^ reported complete outcomes, and 10 studies^[24–32,34]^ reported low reporting bias. The details are shown in Figure 2.

**Table 1:**
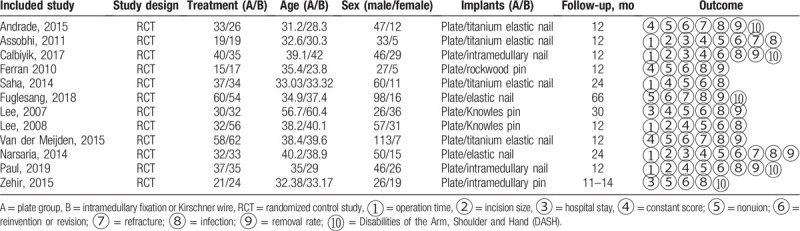
characteristics of included studies.

### 3.2 Shoulder constant and DASH scores

**Figure 3:**
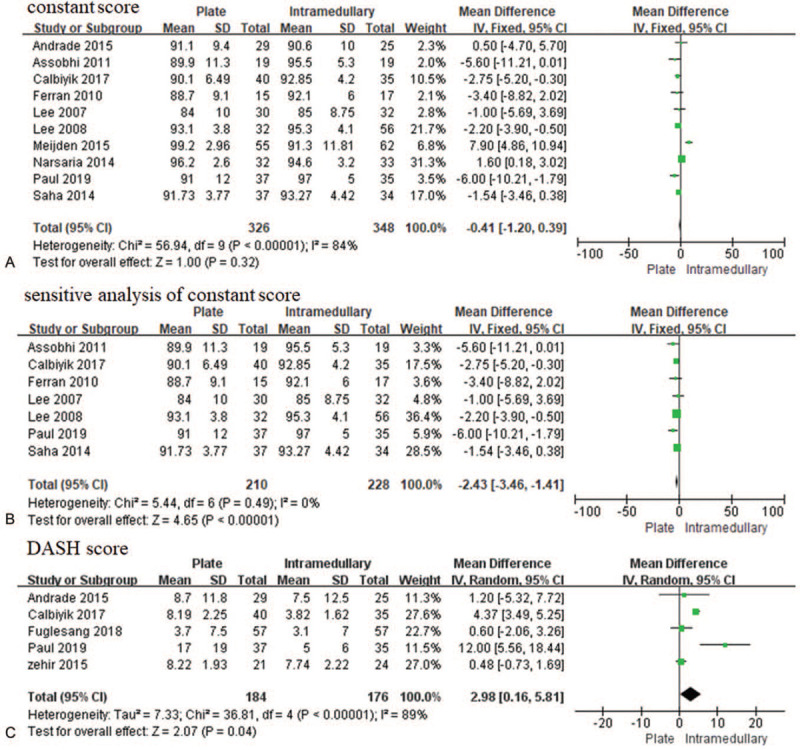
Forest plot for the constant score (A); sensitive analysis of constant score (B); and disabilities of the arm, shoulder, and hand (DASH) score (C). CI = confidence interval, SD = standard deviation.

The shoulder constant score was reported in 10 studies that enrolled 674 patients^[24–29,31–34]^ of whom 326 were treated with plate fixation and the remaining 348 were treated with intramedullary nail/Knowles pin fixation. There was obvious heterogeneity (*I*^2^ = 84%), whereas no significant differences were observed between the 2 methods (*P* = .32) (Fig. 3A). When 3 studies^[24,26,34]^ were excluded from the analysis, the heterogeneity decreased to 0% and a fixed effect model was conducted, which indicated that intramedullary nail/Knowles pin fixation could significantly improve the shoulder constant score significantly [MD = −2.43, 95% CI (−3.46–1.41), *P* < .000 01] (Fig. 3B). The DASH score was reported in 5 studies involving 360 patients,^[23,29,30,32,34]^ and our analysis found that intramedullary nail/Knowles pin fixation showed significantly better shoulder function than plate fixation [MD = 2.98, 95% CI (0.16–5.81), *P* = .04, *I*^2^ = 89%] (Fig. 3C). We conducted sensitivity analysis by eliminating each study one by one while the heterogeneity was stable.

### 3.3 Complications

**Figure 4:**
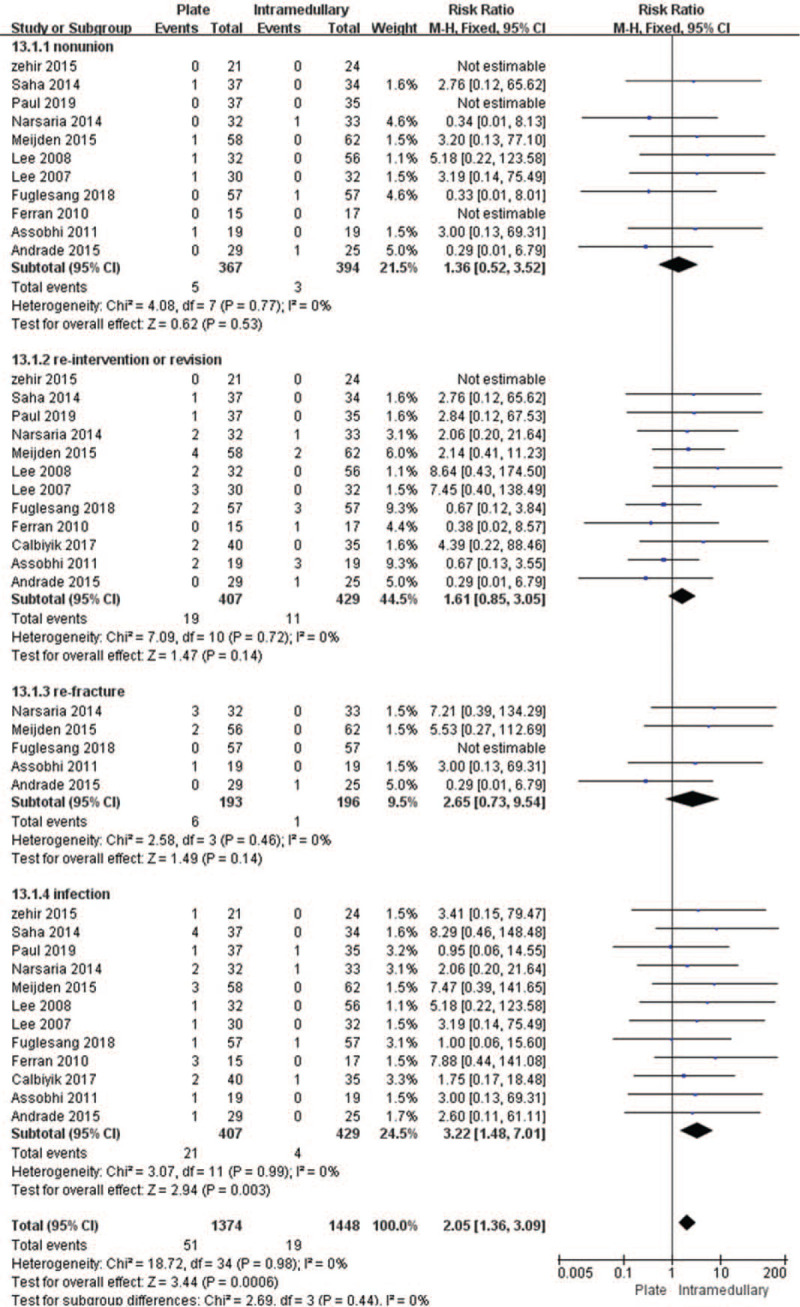
Forest plot for the complications including nonunion, reintervention or revision, refracture, and infection. CI = confidence interval, SD = standard deviation.

**Figure 5:**
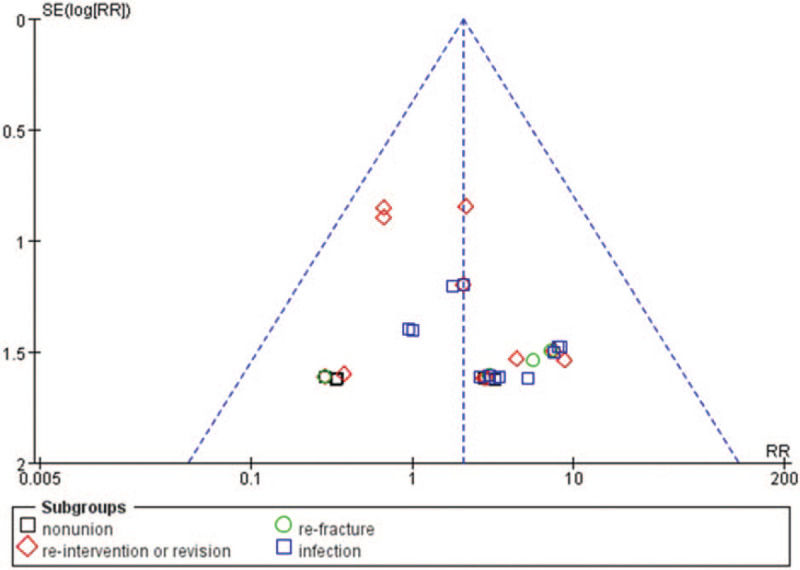
Funnel plot of the publication bias. RR = relative risk.

All studies included in the meta-analysis comprising 2822 fractures (1374 treated with plate and 1448 treated with intramedullary nail/Knowles pin fixation) were analyzed for complications, such as nonunion, reintervention, or revision, refracture, and infection. The fixed effects model was used to analyze the complications associated with both the fixation methods, and our analysis revealed that the incidence rate of complications, particularly infection rates [RR = 3.22, 95% CI (1.48–7.01), *P* = .003, *I*^2^ = 0%] associated with plate fixation, was significantly higher [RR = 2.05, 95% CI (1.36–3.09), *P* = .0006, *I*^2^ = 0%] than those associated with intramedullary nail/Knowles pin fixation. However, there were no significant differences in nonunion (*P* = .53), reintervention or revision (*P* = .14), and refracture (*P* = .14), between these 2 fixation methods (Fig. 4). Although publication bias was detected, there were no significant bias existed (Fig. 5).

### 3.4 Operation time, incision size, and hospital stay

**Figure 6:**
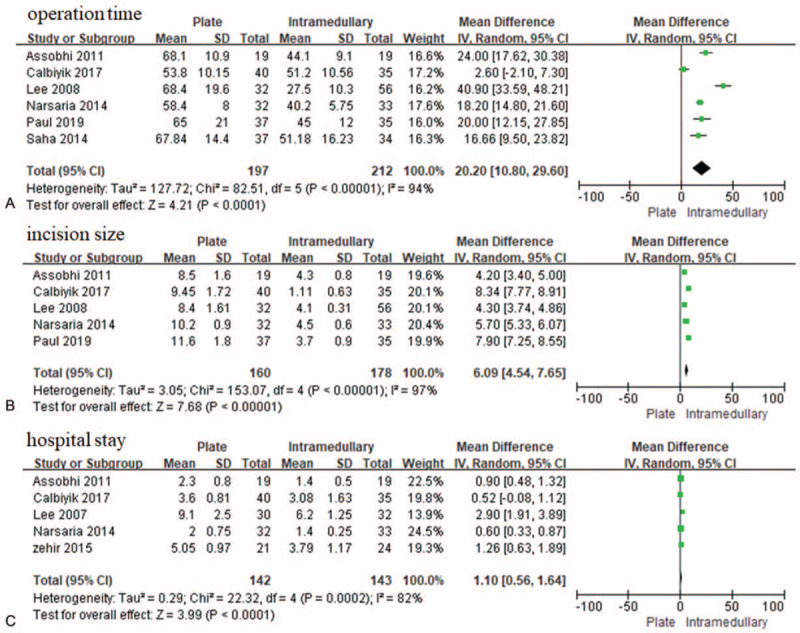
Forest plot for the operation time (A), incision size (B) and hospital stay (C). CI = confidence interval, SD = standard deviation.

Six studies^[25,26,27,29,32,33]^ that enrolled 409 patients reported operation time and concluded that the operation time was longer with plate fixation for displaced midshaft clavicle fracture than with intramedullary nail/Knowles pin fixation [MD = 20.20, 95% CI (10.80–29.60), *P* < .00001, *I*^2^ = 94%] (Fig. 6A). Five studies^[26,28,29,32,33]^ that reported incision size in 338 patients found that intramedullary nail/Knowles pin fixation decreased the incision size significantly [MD = 6.09, 95% CI (4.54–7.65), *P* < .00001, *I*^2^ = 97%] (Fig. 6B). Five studies^[23,26,27,32,33]^ that reported hospital stay in 285 patients found that hospital stay after plate fixation was significantly longer than it was after intramedullary nail/Knowles pin fixation group [MD = 1.10, 95% CI (0.56–1.64), *P* < .0001, *I*^2^ = 82%] (Fig. 6C). We conducted sensitive analysis for operation time, incision size, and hospital stay. For operation time and incision size, we found that the heterogeneity was stable when each study was eliminated one by one. For hospital stay, one study^[27]^ was found to contribute to the heterogeneity. After exclusion of this study, no significant difference was observed between the 2 fixation methods with no heterogeneity [MD 0.73, 95% CI (0.53–0.93), *P* < .00001, *I*^2^ = 36%].

### 3.5 Removal rate

**Figure 7:**
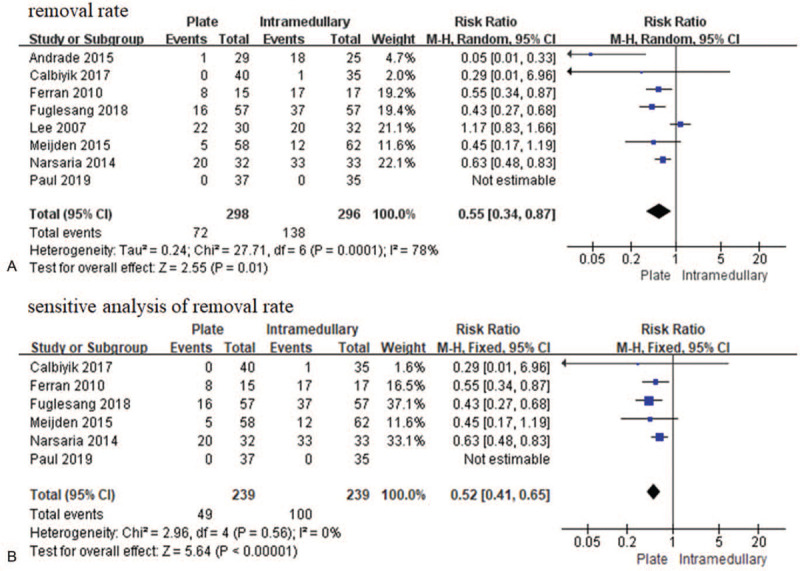
Forest plot of the removal rate (A) and sensitive analysis of removal rate (B). CI = confidence interval, SD = standard deviation.

A second surgery could increase several risks and increase the economic burden on the patient. Removal rates were reported for 594 patients in 8 studies,^[26,27,29–32,34]^ and our analysis showed a higher removal rate with intramedullary nail/Knowles pin fixation than with plate fixation [RR = 0.55, 95% CI (0.34–0.87), *P* = .01, *I*^2^ = 78%] (Fig. 7A). We found 2 studies^[27,34]^ that contributed to the heterogeneity. After exclusion of these studies, we found no significant difference was observed between the 2 fixation methods with no heterogeneity (RR 0.52, 95% CI (0.41–0.65), *P* < .00001, *I*^2^ = 0%) (Fig. 7B).

## 4 Discussion

In this study, we found that compared with plate fixation, intramedullary nail/Knowles pin fixation offered several complications related to operation time, incision size, hospital stay, shoulder constant score, and DASH scores and was associated with a higher removal rate and a fewer infections compared to plate fixation. Nonunion, refracture, and reintervention were comparable between the 2 methods.

Recently, some previous meta-analysis have been reported the comparison of plate versus intramedullary nail//Knowles pin fixation.^[16,19,20,35,36]^ Gao et al^[20]^ reported only 6 RCTs and Duan et al^[17]^ reported only 4 RCTs. Compared with study by Gao et al^[20]^ and Duan et al,^[17]^ our meta-analysis included 12 RCTs. For shoulder constant score, the heterogeneity decreased from 84% to 0% after eliminating 3 studies,^[24,26,34]^ thus improving the shoulder constant score with the intramedullary nail fixation, which is consistent with the findings reported by Zhu et al.^[14]^ For DASH scores, compared with plate fixation, intramedullary nail/Knowles pin fixation resulted in significantly lower scores despite the high heterogeneity (*I*^2^ = 89%). The heterogeneity was stable after eliminating each included study, which may be due to data subjectivity as well as not blinding. However, we found that the DASH score with intramedullary nail/Knowles pin fixation for all the 5 studies was less than that with plate fixation, although without significance, which may be because of the sample size being too small.

In addition to the functional assessment, Surgeons also paid more attention to complications. A review by Barlow et al^[18]^ indicated a trend toward a lower complication rate with intramedullary fixation. However, we found that the major complications included wound infection, nonunion, and implant failures; therefore, we could not determine the details of each type of complications. In our study, we divided complications into nonunion, reintervention or revision, refracture, and infection, which could provide more details for clinicians. Although the incidence rates of nonunion, reintervention or revision, and refracture with the 2 fixation methods are similar and consistent with previously reported incidence rates of nonunion at 12 and 24 weeks^[15]^ and refracture.^[14]^ However, the reason of these complications is not the same in the 2 fixation methods. For plate fixation, the implant failure is mainly caused by excessive movements, which caused the plate to bend or even break. For intramedullary nail/Knowles pin fixation, the lack of stability caused migration of the intramedullary nail/Knowles pin device. The higher infection rate associated with plate fixation versus intramedullary nail fixation in our meta-analysis was consistent with that reported by a previous study.^[19]^ We believe that this is because the plate fixation usually requires a larger incision, wider exposure, more soft tissue dissection, and longer operation time, which increases the incidence of infection. The surgical treatment of displaced midshaft clavicle fractures could be considered a 2-stage process. Intramedullary nail/Knowles pin fixation usually requires a secondary surgery, which is not the case with plate fixation. In our meta-analysis, the number of patients who required implant removal after intramedullary nail/Knowles pin fixation was twice the number of patients who required plate fixation, and this was mainly due to pain from the nail's entry portal, which was related to the protruding nail's instability.^[30]^

Our findings related to operation time, incision size, and hospital stay were consistent with those reported by a previous study.^[20]^ We observed significance in operation time and incision size when a sensitivity analysis was performed by eliminating each study that was included in the meta-analysis, indicating the reliability and validity of our findings. Regarding the hospital stay, when one study^[27]^ was excluded, the heterogeneity decreased from 82% to 36%. We believe that this may be due to different hospital management systems, such that the hospital stay is significantly longer than that reported by other hospitals.

The present study had some strengths. First, compared with previous review studies,^[15,16,37,38]^ the present study enrolled 3 recent studies,^[29,30,32]^ and 12 trials in total were enrolled. Second, the present study had a prospective randomized controlled design with a longer follow-up duration of 12 to 66 months for enrolled trials. Third, the complications were divided into nonunion, reinvention or revision, infection, and refracture, which provided more guidance for surgeons involved in these procedures unlike that in other studies.^[36]^ However, the present study also had some limitations. First, some RCTs included in this study did not demonstrate the random generated details. Second, the duration of follow-up was not consistent in all the included studies and the shoulder function in early stage and late stage was different than that in a previous study.^[39]^ Third, the present meta-analysis enrolled only full-text articles in English, which could lead to selection bias.

Intramedullary nail/Knowles pin fixation could improve shoulder function and have lower infection. Considering the better performance of intramedullary nail/Knowles pin fixation for displaced midshaft clavicle fractures, we recommend this procedure as the first choice for treatment. However, RCTs with good methodology should be performed in the future due to some limitations in the current evidence.

## Author contributions

**Conceptualization:** Xueyang Tang.

**Data curation:** Lang Li, Fei Xing.

**Formal analysis:** Lang Li, Fei Xing.

**Funding acquisition:** Lang Li, Xueyang Tang.

**Investigation:** Fei Xing.

**Methodology:** Lang Li, Fei Xing.

**Project administration:** Xueyang Tang.

**Resources:** Xiaodong Yang, Xueyang Tang.

**Software:** Fei Xing.

**Supervision:** Jun Jiang.

**Writing – original draft:** Lang Li.

**Writing – review & editing:** Lang Li, Xueyang Tang.
